# Plant Extracts as Antimicrobial Agents in Sustainable Conservation of *Erythrina caffra* (Fabaceae) Historical Trees

**DOI:** 10.3390/antibiotics12071098

**Published:** 2023-06-24

**Authors:** Franco Palla, Anahì E. A. Bucchini, Laura Giamperi, Pasquale Marino, Francesco M. Raimondo

**Affiliations:** 1 Department of Biological, Chemical and Pharmaceutical Sciences and Technologies/Section of Botany, Anthropology and Zoology, University of Palermo, Via Archirafi 38, 90123 Palermo, Italy; 2 Botanical Garden, Department of Biomolecular Sciences, University of Urbino, Via Bramante 28, 61029 Urbino, Italy; elena.bucchinianahi@uniurb.it (A.E.A.B.); laura.giamperi@uniurb.it (L.G.); 3 PLANTA/Center for Research, Documentation, and Training, Via Serraglio Vecchio 28, 90123 Palermo, Italy; marino@centroplantapalermo.org (P.M.); raimondo@centroplantapalermo.org (F.M.R.)

**Keywords:** essential oil, hydro-alcoholic extract, antimicrobial activity, natural heritage, urban historical trees, sustainable conservation

## Abstract

Microbial colonization plays a relevant role in the biodegradation and biodeterioration of cultural and natural heritage, representing a revealing problem in conservation strategy. In this study, the essential oil (EO) and hydro-alcoholic extract (HAE) of *Origanum vulgare* L. (Lamiaceae), an aromatic perennial plant, representative of the Mediterranean basin, growing spontaneously and cultivated all over the world, were analysed. Natural products, such as essential oil and hydro-alcoholic extract, have strong antiseptic and antimicrobial properties and are ad hoc applied for the sustainable conservation of *Erithryna caffra* (Fabaceae). The main taxa revealed in the damaging of these arboreal heritage, are *Bacillus* sp., *Streptomyces* sp. and *Terribacillus* sp. (as bacteria), *Alternaria* sp., *Aspergillus* sp. and *Chaetomium* sp. (as fungi). GS-MS analysis identified carvacrol, thymol and their biosynthetic precursors *γ*-terpinene and *p*-cymene, as main components, and the antimicrobial efficiency assayed by in vitro methods (Agar Dish Diffusion, Well Plate Diffusion). In this study, by combining the application/exposure of both HAE and EO, the bacterial and fungal colonies development has been in vitro countered. The results confirm the possible use of plant products as a valid alternative to the traditional synthetic chemical biocides, with full respect to the environment.

## 1. Introduction

**Figure 1:**
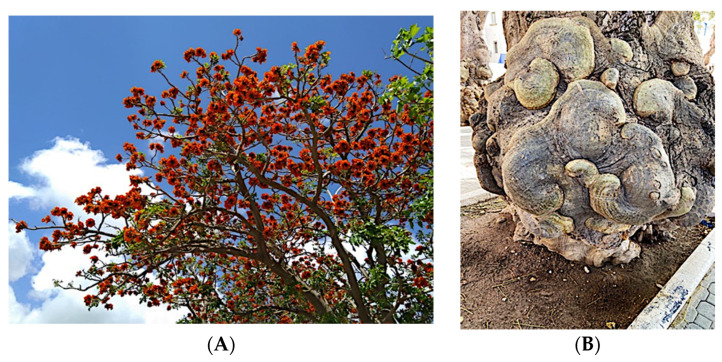
(**A**) Flower of *Erithrina caffra*, the colour resembles the coral; (**B**) example of the peculiar morphology of *Erythrina caffra* trunk.

Significant roles, in requalifying and revitalizing city areas, are attributable to peculiar trees such as *Erythrina caffra* Thunb. (Fabaceae), originally from South Africa, introduced in the city centre of Trapani (Italy) because “coral tree”, the symbol of coral craftsmanship active in the Trapani area [1]. Peculiar of these trees are the flowers (Figure 1A), resembling the red colour of Mediterranean coral, and the trunks (Figure 1B). In some of these historic and symbolic trees, bio-deteriorated condition has been revealed, particularly in some inner parts of the trunk colonized by microbial systems. Biological wood decay is mainly related to bacterial, fungal colonisation and insect infestation, able to hydrolyse the chemical constituents of wood (cellulose, hemicelluloses, lignin). Although different degradation steps can be distinguished in relation to woody plant species and the conservation strategies, a relevant role is played by temperature, relative humidity and lighting [2]. Conventional treatments to control these processes provide for the use of synthetic chemical biocides (SCB), whose toxicity for humans and environments is well known, as well as their persistence in natural environments, also contaminating areas far from the site of treatment [3,4]. A valid alternative to SCB has already been represented by bioactive molecules extracted from plant matrices and in particular from aromatic ones. These natural products, essential oils (EOs) and hydro-alcoholic extracts (HAEs) have been used as antimicrobial, antioxidant, and pesticidal agents in several fields, including agriculture, cosmetics, food, medicine, and pharmacy, from ancient times and becoming source of new drugs [5,6,7,8,9,10]. In the last decades, the use of these natural products is increasingly indispensable, both for environmental and human safety and in reducing the microbial resistance phenomena [11,12,13]. Nowadays, plant products have been successfully applied to perform green conservation strategies of cultural assets able to counteract microbial deterioration, induced by fungi and bacteria, by inhibiting the growth and metabolic activity of these microorganisms without undesirably affecting the substrates [14,15,16]. While chemical synthetic biocides are easy to apply, they do not have single biological targets and can induce several negative side effects such as persistent residues in the environment, which are detrimental to human health. Although the use of commercial biocides has been performed for many years, there is still no detailed information on their effectiveness over time [17,18]. Furthermore, the choice of a biocide is restricted by the Directive 98/8/EC (European Parliament and Council—1998L0008-EN-26.09.2008-009.0019). Based on the antimicrobial properties of Lamiaceae plants’ essential oils (EO) and on previous our studies [19,20,21,22,23] aromatic plant extracts (EO and HAE) have been utilized to counteract the biodeterioration on the *E. craffa* historical tree. Specifically, the exposure to the volatile compounds of *Origanum vulgare* L essential oil has been preceded by the direct application of the corresponding hydro-alcoholic extract. These extracts mainly contain phenolic monoterpenes such as carvacrol and thymol, and their biosynthetic precursors *γ*-terpinene and *p*-cymene. Gas Chromatography/Mass Spectrometry (GC-MS) analysis reveals the presence of several phenolic monoterpenes, with well-known anti-inflammatory and anticancer properties such as thymol and carvacrol, respectively 27.18% and 4.04% in EO, 0.05% and 0.04% in HAE.

The antimicrobial activity of both *O. vulgare* L. extracts against the bacterial and fungal colonies, isolated from degraded wood fragments collected inside the tree trunk, has been in vitro tested by the Agar Diffusion Disc and Well Plate Diffusion methods [6,20].

In our hypothesis, combining a preliminary application of HAE with exposure to the volatile compounds of the corresponding EO, the antimicrobial action is enhanced, as reported in our previous study on a wooden sculpture [24].

In this study, for the first time, plant products, such as EO and HAE, are in combination applied to set up a strategy for the sustainable conservation of historical trees. Furthermore, considering the impact (close to zero) on human health and the environment, this protocol can be repeated over the time, avoiding the harmful effects induced by synthetic chemical biocides.

## 2. Results

### 2.1. Inhibition Microbial Growth Assays

The antimicrobial activity of *O. vulgare* extracts (50% EO and 100% HAE) has been evaluated by two in vitro methods, Agar Disc Diffusion and Well Plate Diffusion, different growth inhibition halos were revealed and summarized in Table 1. The inhibition halo diameters (i.h.d.) are closely related to EO or HAE and the different microbial taxa. Inhibition halos were measured, assessing the antimicrobial activity as positive (i.h.d. > 9 mm), moderate (i.h.d. between 9 and 6 mm) and low (i.h.d. < 6 mm).

**Figure 2:**
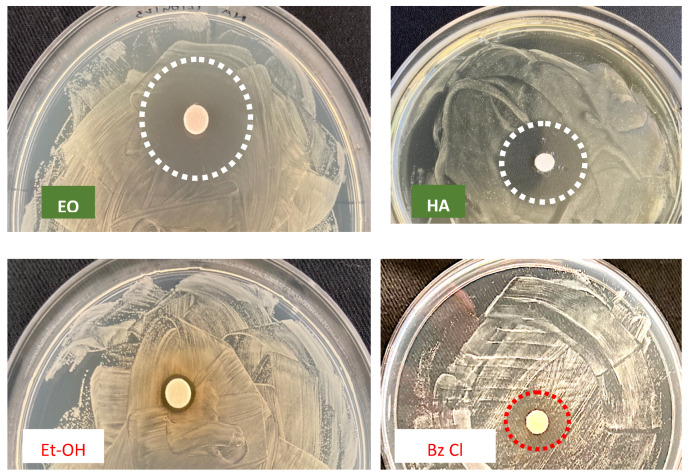
ADD assay vs. *Bacillus* sp., to evaluate the antimicrobial activity of *O. vulgare* products: EO = 50% essential oil and HAE = 100% hydro-alcoholic extract, in comparison with the Control solutions: Et-OH = 70% Ethanol and Bz Cl = 3% *v*/*v* Benzalkonium Chloride. Dotted circles = growth inhibition halo; barely detectable for Et-OH.

**Figure 3:**
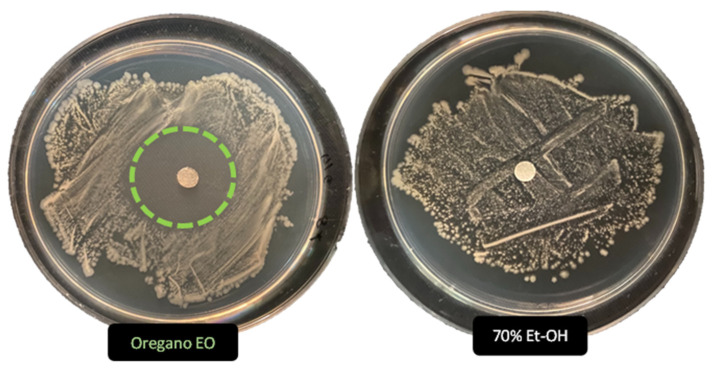
Antimicrobial activity (ADD assays) of *O. vulgare* essential oil (50%) and the Ethanol control solution (70%) vs. Streptomyces sp. Dotted circle highlight the noticeable activity of Oregano EO, undetectable for 70% Et-OH solution.

The size of inhibition halos was different in relation to bacterial or fungal strains. As shown in Figure 2 and Figure 3 the ADD assays reveal a different antimicrobial activity: higher for the 50% EO solution (i.h.d. > 9 mm) and moderate (i.h. d. > 6 mm) for the 100% HAE. Control solutions were the commercial biocide 3% Benzalkonium Chloride and 70% Ethanol, producing respectively inhibition halos <6 mm or so reduced to be difficult to detect. 

Differences in the diameter of inhibition halos are attributable to the chemical composition of the plant products (particularly to carvacrol and thymol concentration) and the microbial cell structures. These plant extracts can act on the cell wall structure or lipid/protein composition of cytoplasmic membranes, or influencing specific physiological processes [25,26,27,28,29].

### 2.2. Exposure to EO Volatile Compounds

Hypothesizing the enhancement of the antimicrobial action of *O. vulgare* essential oil when combined with the related hydro-alcoholic extract, colonized/degraded fragments of inner zones of *E. caffra* trunk, were used in vitro essays, carried out with these plant products. The exposure was performed in glass Petri dishes (6 cm in diameter), sealed by parafilm and keeping them in a glass desiccator for the experimental time of four weeks. 

**Figure 4:**
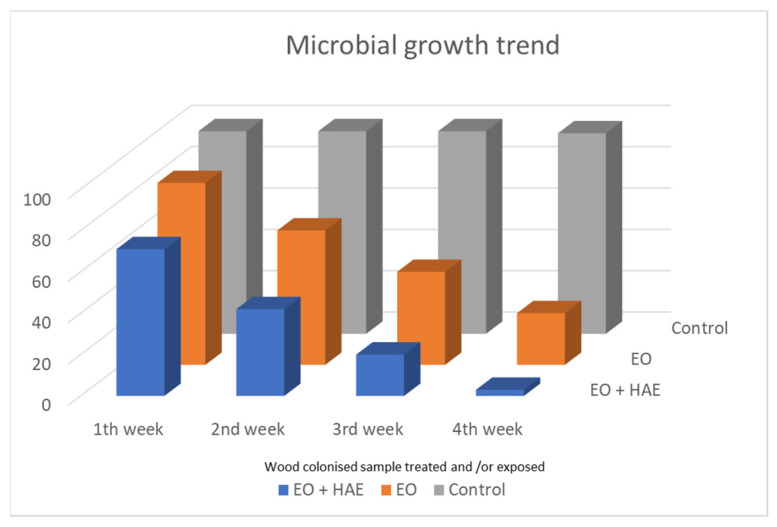
Monitoring of microbial load during the four weeks of exposure. Wood colonized fragments were exposed to only EO volatile compounds or after a preliminary application of the related HAE (EO + HAE); solutions were *O. vulgare* 50% EO and 100% HAE. In Control samples, the colonized wood fragments were kept in the equal exposure condition without any plant product.

Antimicrobial assays were performed using wood decaying fragments (about 2 × 4 × 2 cm), applying the HAE directly on fragments surface first, and subsequently exposing them to volatile compounds of the Essential Oil (Figure 4, EO + HAE). The result was compared to effects only of the exposure to the Essential Oil volatile compounds (Figure 4, EO); control samples were performed keeping wood colonized fragments in equal exposure conditions without the presence of plant extract (Figure 4, Control). 

Thermo-hygrometric parameters were constant (Temp. 20 + 2 °C, R.H. 60 + 2%).

### 2.3. Microbial Load Monitoring

During the four weeks of exposure in the dedicated glass system, the monitoring of microbial load on colonized wood fragments was carried out weekly. Each week an aliquot of the wood-exposed fragments (EO or EO + HAE) has been picked up, and subsequently utilized to inoculate Nutrient agar plates. As Control, wood colonized fragments have been kept for the same period (four weeks) in the exposure system, without any *Origanum vulgare* extracts.

In Figure 4 the results of microbial load, during the four weeks of monitoring, are shown. If no *O. vulgare* derivative is present, a high microbial load was revealed in all control samples (C, grey histogram columns). The microbial load is clearly affected by the exposure to only *O. vulgare* EO, and it is almost completely countered when HAE application and EO exposure are combined.

### 2.4. Microscopy Observation

**Figure 5:**
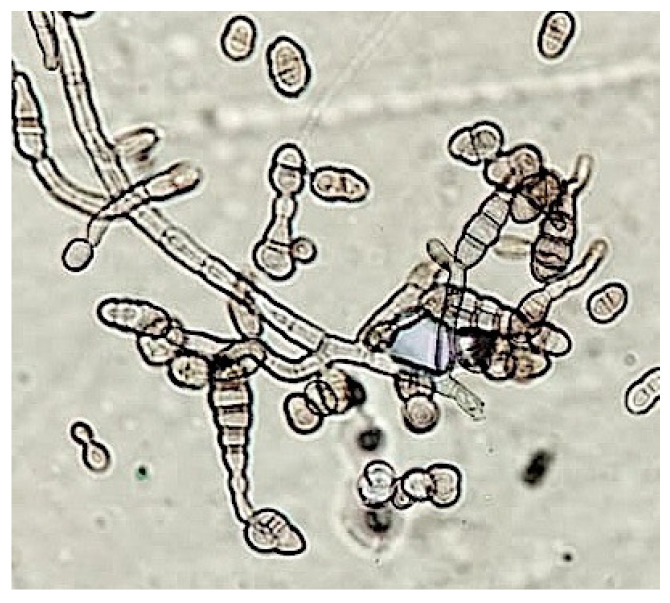
Optical Microscopy observation, reproductive structures of *Alternaria* sp. are clearly detectable, after staining by Lugol’s reactive (magnification equal to 40×).

**Figure 6:**
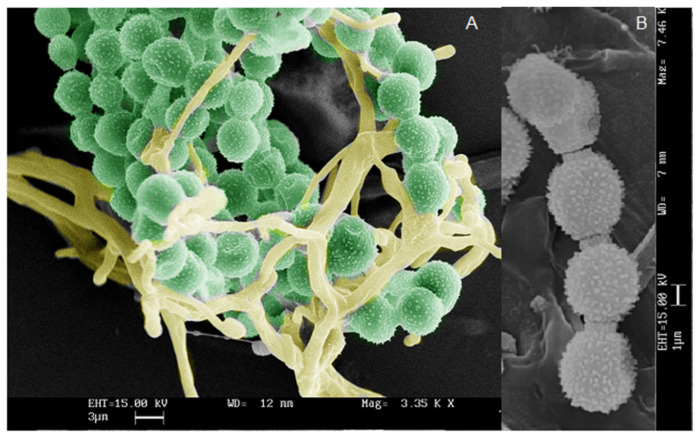
Scanning Electron Micrography of *Aspergillus* sp. reproductive structures: (**A**) conidia and hyphae; (**B**) magnified detail of conidia. Bar in the micrographs corresponds to 3 (**A**) and 1 (**B**) micrometer respectively.

Microscopy analysis has been performed by Optical (OM) and Scanning Electron Microscope (SEM). Fungal reproductive structures were observed, after Lugol’s iodine staining, by optical microscope, and the peculiar profile of spores allows to correlate them to *Alteraria* sp., Figure 5. SEM micrographs in Figure 6, showed *Aspergillus* sp. reproductive structures, in Figure 6A hyphae and conidia structure have been coloured, using GIMP (GNU Image manipulator), instead a magnified detail of conidia is shown in Figure 6B.

## 3. Discussion

A characteristic of aromatic plants is the presence of complex mixture of aliphatic, aromatic and terpene compounds, that generally have a powerful role in plant interaction and pollinator attraction [30,31]. Their extracts (EO, HAE) have aromatic components, which give distinctive flavour, odour or scent to each plant. Mixtures of low-molecular-weight terpenes (hydrocarbons) and terpenoids (oxygen-containing hydrocarbons) such as acids and esters, acyclic monoterpene alcohols, aliphatic aldehydes, aromatic phenols, monocyclic and bicyclic alcohols, monocyclic ketones, bicyclic monoterpene ketones are included. Some essential oils can also contain oxides, sulphur- containing constituents, methyl anthranilate, coumarins, and some other chemical compounds. 

The peculiar action of *Lamiaceae* plant essential oils, as well as their hydro-alcoholic extracts, in counteracting the growth of several microbial taxa, have been described in several scientific papers, concerning sustainable approaches in several fields (agriculture, food storage and wood industries) [32,33,34,35,36,37].

Furthermore, these bioactive natural products have fruitfully been proposed in several green protocols for the sustainable conservation of cultural assets, as also reported in our previous investigations [20,21,22].

Plants from Asteraceae, Lamiaceae, Rutaceae, Myrtaceae, and Zingiberaceae families are an excellent source of bioactive products [38]. The EO of *Cinnamomun zeylanicum* and the hydrolate of *Citrus aurantium* have been used as emulsion in a green restoration protocol of modern painting [39]. A strong and synergic antimicrobial effect has been highlighted using a mixture of different EOs (basil, cloves, thyme, pine, and tea trees) in comparing to a synthetic chemical biocide (ammonium quaternary salts) [40]. In conservation action of archaeological wood, Eos from *Cinnamomum zeylanicum*, *Thymus serpyllum,* and *T. vulgaris* were assessed to counteract microbial wood biodeterioration; a significant decrease of fungi growth has been reported [41]. Fungal growth *(Aspergillus fumigatus, Cladosporium cladosporoides*, *Penicillium chrysogenum*) revealed on paper substrata has been inhibited using a mixture of lemongrass, oregano, and peppermint (ratio 1:1:1) essential oils [42]. In a recent review [43] applications of these plant products and their antimicrobial activities are issued, highlighting their use on inorganic and organic cultural objects.

Finally, but not least, the human risk assessment considers the protection of operator health, in relation to the use of synthetic chemical biocides. The risk is often frequently underestimated, requiring the evaluation of both, the microbial taxa characteristics and bioactive compounds in the plant products, to perform adequate antimicrobial treatments.

## 4. Materials and Methods

### 4.1. Sampling Inside the Tree Trunk

**Figure 7:**
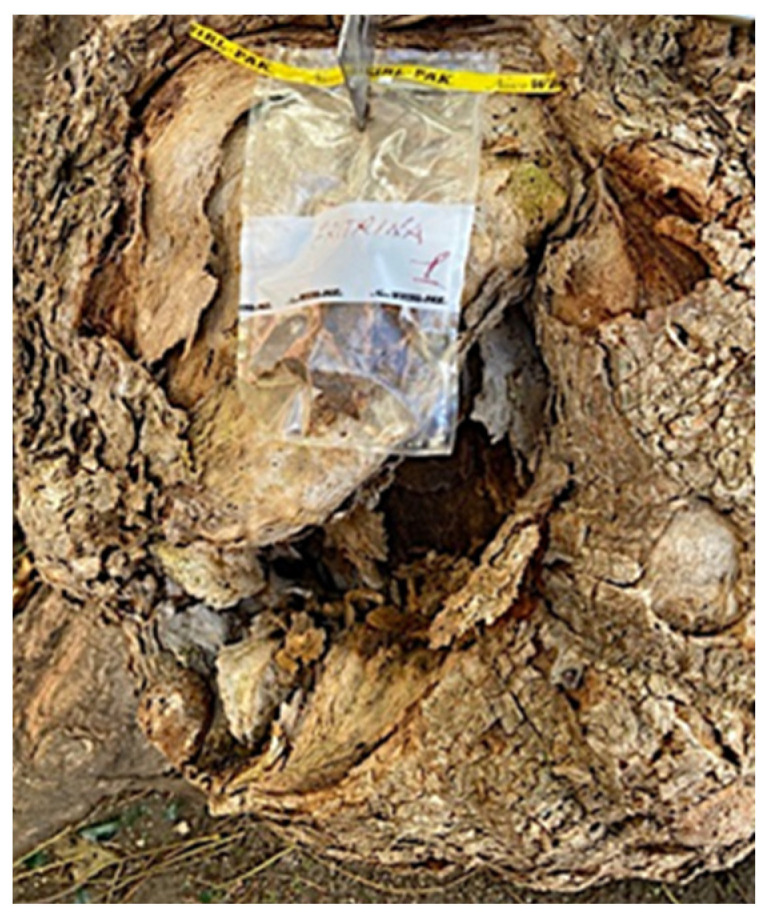
Aliquots of deteriorated/colonized structures were collected from the trunk cavity and stored in a forensic bag until laboratory assays.

Aliquots of deteriorate/colonized areas of the inner part of the trunk were collected and stored in a forensic bag, avoiding undesirable contamination (Figure 7), until use in the laboratory.

### 4.2. The Definition of Taxa

The wood’s structural properties are related to several components, mainly cellulose, hemicellulose and lignin, but other molecules such as pectin, proteins, simple sugar, and starch are also present; all those can become nutrient sources for bacteria and fungi colonies. In fungi decay, the depolymerization of cellulose, hemicellulose or lignin wood components is recognizable as brown, white or soft rot, with an evident loss of mechanical properties. The deterioration processes are certainly related to the metabolism of microbial taxa but are deeply influenced by environmental conditions [36,37]. In this study, fragments from a deteriorated area of *Erythrina caffra* trunk have been utilized to inoculate Nutrient Agar Plate (Difco), and after incubation at 30 °C for 16–48 h, bacterial and fungal colonies have been isolated. Concerning the taxonomical identification, the 16S and ITS target rDNA sequences have been utilized for the identification respectively of bacterial and fungal taxa, in PCR reactions [44,45,46]. PCR products were previously purified by PCR Purification Kit (PureLink, Thermo-Fisher, Milano, Italy) and sequenced by Sanger method, utilizing Eurofins service (MWG Genomics, Ebersberg, Germany, based on Sanger method). Sequences analysis (% of similarity) was performed by the BLAST Platform, referring to the EMBL (Germany) and NIH (USA) genomic databases [47]. The prevalent genera were *Bacillus*, *Streptomyces*, and *Terribacillus* (all Gram+ bacteria) and *Alternaria*, *Aspergillus*, and *Chaetomium* (fungi).

### 4.3. Plant Extracts

The biocide activity of both *O. vulgare* EO and HAE is related to their chemical compounds. Concerning essential oil (100% pure essence, doTerra Europe, doterra.com) the principal components have been identified by Gas Chromatography–Mass Spectrometry. Large amounts of phenolic monoterpenes have been identified (related to the cymyl-pathway), highlighting the presence of thymol, carvacrol, and related precursors (*p*-cymene and *γ*-terpinene). Specifically, the Adams, FFSNC2, NIST 11, Wiley 9 databases [48] have been utilized for the identification of chemical compounds, confirmed by the linear retention indices (SciFinder database); identification completed by with laboratory standards (STEBICEF—Laboratory of Natural Products). In Table 2 the principal compounds are reported. The hydro-alcoholic solution was provided by EPO Srl (Milano, Italy), and its content in thymol (0.05%) and carvacrol (0.04%) have been determined by Gas-chromatography, according to the European Council Pharmacopoeia [49].

### 4.4. In Vitro Assays of Antimicrobial Activity 

The antimicrobial activity of the *O. vulgare* extracts (EO and HAE) was assayed by Agar Diffusion Disc (ADD) or Well Plate Diffusion (WPD) methods. Microbial liquid cultures were performed at 30 °C (incubation for 16–48 h), normalizing the concentration of 1 × 10^6^ CFU/mL (bacteria) and 1 × 10^4^ conidia/mL (fungi). To perform the inhibition growth assays, 20 μL of each liquid culture was uniformly spread, by a disposable sterile Drigalsky spatula, on the surface of agar media incubating at 30 °C for 60 min; allowing the surface to dry. Each assay has been performed in triplicate.

In the ADD assay, 15 μL aliquots of *O. vulgare* EO (50% solution) were dropped on a sterile 6 mm in diameter paper disc (Dutscher paper, Issy-les-Moulineaux, France), centred on a Nutrient agar surface (Petri dishes, 9 cm in diameter), previously sown with a microbial culture. After incubation at 30 °C inhibition halos (I.H.) were clearly revealed, instead confluent microbial growth was observed in the remainder of the agar surface. The inhibition halo diameter (mm) of I.H. is strictly related to the antimicrobial activity of *O. vulgare* essential oil vs. each microbial taxon. Proper controls were performed by soaking, the paper disc with 70% Ethanol or 3% (*v*/*v*) Benzalkonium Chloride solutions.

In the WPD assay, a hole of about 4 mm in diameter was aseptically drilled on the agar by a sterile glass capillary. Each hole was filled with 15 μL of *O. vulgare* HAE and the plate was incubated at 30 ± 1 °C, for up to 48 h. Growth inhibition halos, different in diameter, were observed and measured (mm).

Distinguishing minimum inhibitory concentration (MIC) and minimum biocidal concentration (MBC), the biocide or biostatic activity was also valued. Specifically, MIC corresponds to any visible microbial growth, incubating at 30 °C, with the lowest biocidal concentration. Instead, MBC corresponds to the lowest concentration of essential oil solution (100%, 50%, 25%) able to kill 99.5% of the original inoculum.

These microdilution assays were performed in 96-well microliters plates (Kartell), and in each well were mixed 30 μL of EO solution; 30 μL of microbial culture and 30 μL of nutritive broth. The microbial growth (18–36 h of incubation at 30 °C) was assessing the absorbance (O.D.) values at 500–600 nm; the commercial biocide Benzalkonium Chloride (3%, *v*/*v*) was utilized as control [20,50]. 

### 4.5. Microscopy Observation

Performing Optical and Scanning Electron Microscopy observations, the profile of fungal conidia and hyphae were clearly distinguished. Specifically, fungal structures were sampled in the upper part of isolated colonies by adhesive tape strip (fungi tape, DID) and immediately gently placed on sterile glass slide for microscopy. After staining with Lugol’s reactive, by Optical Microscope (Leica, Wetzlar, Germany, 40× magnitude), as in Figure 5. 

SEM analysis were performed using the aluminium STUB (Assing), equipped with adhesive tape. The fungal structures, gently sampled from the fungal colonies, were covered with gold particles (Agar Auto Sputter Coater) and observed under vacuum conditions (Leica Leo—420). SEM analysis allows the observation of fungal structure, as shown in Figure 6, related to *Aspergillus* sp. colonies. Conidia and hyphae (Figure 6A) have been highlighted by artificially colouring, using the GIMP (GNU Image manipulator). Furthermore, magnified detail of conidia is shown in Figure 6B.

## 5. Conclusions

The present work aims to define green strategy to preserve natural heritage through an eco-friendly procedure. Specifically, *Erythrina caffra* historical trees have been the target in counteracting the biodeterioration processes induced by microbial colonization in the inner part of the tree trunk. As reported in the literature, Ascomycota species can induce a decay of wood substrate causing the “soft rot” of wood. In the wood-decayed samples from *E. caffra,* fungal colonies related to *Alternaria* sp., *Aspergillus* sp. and *Chetomium* sp. have been isolated and identified.

The presence of bacterial colonies was also revealed in the decayed wood sample, identifying *Bacillus* sp., *Streptomyces* sp., and *Terribacillus* sp. as prevalent. As known, these Gram+ bacteria are sensitive to several plant essential oil [43], and for *Streptomyces* sp. colonies even to *O. vulgare* essential oil, as in Figure 4.

In order to counteract this microbial consortium, the combined use of both plant essential oil and hydro-alcoholic extract has been carried out, based on previously conservative procedures successfully gathered on wooden cultural heritage [24]. 

For the first time, *O. vulgare* extracts (EO and HAE) were synergistically applied hypothesizing an improvement in the total antimicrobial activity and defining innovative protocols in the conservation of historical trees. Consequently, ADD and WPD in vitro assays highlighted a more than positive antimicrobial activity for the essential oil and a moderate one for the related hydro-alcoholic extract. 

The results of this study complete and agree with the use of plant extracts as a substitute for synthetic chemical biocides, adding more information for a possible standardization of eco-sustainable conservation approaches. 

Furthermore, their use is increasingly indispensable both for human and environmental safety and in reducing the microbial resistance phenomena [11,51,52,53].

Although it has so far been published a huge number of scientific papers, further information is needed on the possible antagonist/synergic effect of plant products vs. microbial growth; it is extremely hard to predict since each EO is a complex mixture of different chemical compounds [54].

## Figures and Tables

**Table 1:** Antimicrobial activity of the *Origanum vulgare* essential oil (EO) and hydro-alcoholic (HAE) plant extracts, and Control solution (Bz Cl and Et-OH) vs. bacterial and fungal taxa. The average inhibition halos diameter in mm is related to duplicate assays performed by Agar Disch Diffusion or Well Plate Diffusion method.

Microbial Taxa	Inhibition Halo (mm) *O. vulgare*		Inhibition Halo (mm) Control Solutions	
	EO	HAE	*BzCl*	*Ethanol*
	ADD	WPD	ADD	
*Bacteria*	*50%*	*100%*	*3% v/v*	*70%*
*Bacillus* sp.	22 ± 2	9 ± 2	6 ± 3	1 ± 0.5
*Streptomyces* sp.	21 ± 2	6 ± 2		
*Terribacillus* sp.	17 ± 2	5 ± 2		
*Fungi*				
*Alternaria* sp.	16 ± 2	4 ± 2		
*Aspergillus* sp.	13 ± 2	6 ± 2		
*Chaetomium* sp.	15 ± 2	5 ± 2		

**Table 2:** *Origanum vulgare* Essential Oil main chemical compounds (Conc. >1%). Linear Retention Index: (a) on HP-5 MS column; (b) on Supelcowax 10 capillary GC Colum.

LRI (a)	LRI (b)	%	Compound	
1302	2174	27.18	**Thymol**	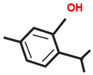
1029	1255	18.97	*p*-Cymene	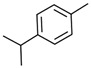
1061	1248	4.50	γ-Terpinene	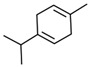
1305	2194	4.04	**Carvacrol**	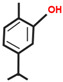
1086	1534	2.80	β-linalool	
927	1028	1.75	α-pinene	
1195	1690	1.34	α-terpineol	
1584	2020	2.00	cariophyllene oxide	
1456	1653	3.60	α-caryophyllene	
1397	1579	5.90	β-caryophyllene	

## Data Availability

The data presented in this study are available on request from the corresponding author.
